# Identification of X chromatin is modulated by complementary pathways in *Drosophila melanogaster*

**DOI:** 10.1093/g3journal/jkae057

**Published:** 2024-03-16

**Authors:** Reem Makki, Victoria H Meller

**Affiliations:** Department of Biological Sciences, Wayne State University, 5047 Gullen Mall, Detroit, MI 48202, USA; Department of Biological Sciences, Wayne State University, 5047 Gullen Mall, Detroit, MI 48202, USA

**Keywords:** dosage compensation, *Drosophila melanogaster*, small RNA, 1.688^X^ satellite repeats, siRNA, *roX*, chromatin entry sites

## Abstract

*Drosophila melanogaster* males have one X chromosome while females have two. This creates an imbalance in X:A gene dosage between the sexes. This imbalance is corrected by increasing transcription from male X-linked genes approximately 2-fold. This process involves the Male-Specific Lethal (MSL) complex, which is recruited to Chromatin Entry Sites (CES) and transcribed X-linked genes, where it modifies chromatin to increase expression. Repetitive sequences strikingly enriched in X euchromatin, the 1.688^X^ satellite repeats, also promote recruitment of the MSL complex to nearby genes. Unlike CES, the 1.688^X^ repeats do not recruit the MSL complex directly. The genetic architecture of recruitment by these DNA elements remains speculative. To facilitate dissection of the mechanism of recruitment, we developed a luciferase reporter system for recruitment of compensation to an autosome. The system was validated by knock down of genes known to participate in compensation. Knock down of factors genetically linked to X recognition reveals that 1.688^X^ repeats recruit through a different mechanism than the CES. Our findings suggest that 1.688^X^ repeats play a larger role during embryogenesis, whereas the contribution of 1.688^X^ repeats and CES is equivalent later in development. Our studies also reveal unexpected complexity and potential interdependence of recruiting elements.

## Introduction

Gene regulation is typically thought of in the context of individual genes but coordinated regulation of large chromosomal domains is also important. An example of this is X chromosome dosage compensation, a crucial process that balances gene expression between males (XY) and females (XX) (Disteche 2012). Dosage compensation is remarkable in that regulatory complexes are selectively recruited to thousands of genes whose only obvious similarity is that they reside on the same chromosome. In both mammals and *Drosophila*, males have one X chromosome while females have two. This creates a potentially lethal imbalance in X to autosome gene expression between the sexes. To maintain an appropriate expression ratio, X-linked gene expression is modulated at the chromosome-wide level. In mammals, dosage compensation is achieved by inactivation of one of the two female X chromosomes (Brockdorff and Turner 2015). In comparison, male fruit flies increase transcription from their X-linked genes approximately 2-fold (Lucchesi et al. 2005; Lucchesi and Kuroda 2015). In both cases, failure to achieve compensation is lethal to the sex that normally modulates expression.

Dosage compensation in *D. melanogaster* involves the action of a ribonucleoprotein complex, formed only in males, called the Male-Specific Lethal (MSL) complex (Gelbart and Kuroda 2009). The MSL complex is composed of five proteins: MSL-1, -2, and -3 (Male-Specific Lethal-1, -2, and -3), MLE (Maleless), and MOF (Males absent On the First). MOF is a histone acetyl transferase that modifies histone H4 at lysine 16 (H4K16) (Smith et al. 2000). This mark is enriched on the X and is associated with increased transcription (Akhtar and Becker 2000). The complex also contains one of two long noncoding RNAs called *roX1* and *roX2* (*RNA on the X-1 and -2*) (Meller et al. 1997; Ilik et al. 2013). *roX1* and *roX2* are encoded on the X chromosome, differ in size and sequence, but functionally redundant. Simultaneous mutation of both *roX* genes disrupts localization of the MSL proteins and causes male lethality (Meller and Rattner 2002). How the MSL complex selectively recognizes X chromatin is still not clearly understood, but the *roX* genes appear to play a critical role in X recognition.

The assembly of the MSL complex is thought to initially occur at sites of *roX* transcription (Kelley et al. 1999; Park et al. 2002; Oh et al. 2003). This is followed by binding of the intact complex to Chromatin Entry Sites (CES). The CES contain a GA-rich motif called the MSL Recognition Element (MRE). This motif is bound by the CLAMP protein, which recruits the MSL complex to the CES (Alekseyenko et al. 2008; Straub et al. 2008; Soruco et al. 2013). The MSL complex then spreads in *cis* to nearby transcribed genes by recognition of the co-transcriptional H3K36me3 mark (Alekseyenko et al. 2006; Larschan et al. 2007; Bell et al. 2008, Sural et al. 2008). This model for recruitment and spreading of the MSL complex remains incomplete. MREs are also present on autosomes and are only modestly enriched on the X chromosome. In addition, CLAMP binds at autosomal MREs, but does not recruit the MSL proteins to autosomes.

Both *roX* genes are X-linked and have internal CES and, when inserted on an autosome, recruit compensation to the insertion site and to active genes nearby (Kelley et al. 1999; Henry et al. 2001; Park et al. 2010). However, *roX* RNA from an autosomal transgene will assemble with the MSL proteins and localize to the X chromosome and rescue *roX1 roX2* males. This demonstrates that the *roX* genes do not confer X-identity (Meller and Rattner 2002). Additional mechanisms must explain the exclusive identification of the X chromosome.

Our lab previously demonstrated that the siRNA pathway plays a role in X recognition (Menon and Meller 2012). A strong candidate for the source of siRNA is a family of 359 bp repeats called the 1.688^X^ satellite repeats (Waring and Pollack 1987; DeBartolomeis et al. 1992; Kuhn et al. 2012). These are strikingly enriched on the X chromosome and dissimilar in sequence to the MREs. When placed on an autosome, 1.688^X^ DNA attracts compensation to nearby genes (Joshi and Meller 2017; Deshpande and Meller 2018). Some 1.688^X^ repeats are transcribed and produce siRNA (Aravin et al. 2003; Menon et al. 2014). Ectopic expression of siRNA from the 1.688^3F^ repeat (at cytological position 3F) partially rescues *roX1 roX2* mutant males (Menon et al. 2014). This suggests that the 1.688^X^ satellite repeats play a role in X recognition. Significantly, recruitment of compensation in *cis* to autosomal 1.688^X^ insertions was enhanced by 1.688^3F^ siRNA, but recruitment by an autosomal *roX1* insertion was not, suggesting that the 1.688^X^ repeats and *roX* genes with CES recruit through different pathways (Joshi and Meller 2017). As a first step towards understanding how these elements function, we developed a system to test candidate genes for a role in recruitment by each element.

The redundant function of recruiting elements and the presence of hundreds of 1.688^X^ repeats on the X chromosome makes it impossible to evaluate their function in the context of the X. We developed a dual luciferase system to detect recruitment by individual elements inserted on an autosome. We validated this system by knock down of genes known to be involved in dosage compensation. We then examined genes that enhance the male lethality of partial loss of function *roX1 roX2* mutants as these might further reduce association of the MSL complex with the X chromosome, implying a role in X recognition. We find that *CLAMP* knock down selectively blocks recruitment by the CES-containing *roX1* and knock down of *Ago2* and *Su(var)3-7*, genes that function as small RNA effectors and in the formation of heterochromatin, respectively, disrupt recruitment by 1.688^X^ repeats but not *roX1*. These findings confirm that the mechanisms of recruitment by *roX1* and 1.688^X^ repeats are different. Our studies also reveal unexpected complexity and potential interdependence of recruiting elements. This system can be used to screen any candidate gene with a potential role in recruitment, and it can be adapted to test the ability of other DNA elements to recruit compensation.

## Materials and methods

### Creation of firefly luciferase reporter

A construct containing the Firefly luciferase coding sequence (Fluc) flanked by the 5′ and 3′ untranslated regions (UTRs) of *msl2* is described by Gebauer et al. (1999). The SV40 terminator was amplified from pAc5.1C-Fluc-V5His6 (Addgene) using primers SV40 F1 (ACGTGGATCCGACATGATAAGATACATTGATGAG) and SV40 R1 (ATATGGATCCGGTACCTCGAGTGAAACATAAAATGAATG), digested with Bam H1 and inserted into a Bgl II site flanking the 3′ UTR to create mLm-SV40. A Kpn 1 site was introduced at the 5′ end of mLm-SV40 by amplification with primers mLm F1 (AGTAGAGCTCGGTACCGCCCAATTCTTCCTTTGACGG) and mLm R1 (TGCCTCCTGGGCTAGTTACCTG) and replacement of a Sac 1 fragment in mLm-SV40 with the digested amplicon. A 304 bp Nco 1 - Kpn 1 fragment of pAc5.1C-Fluc-V5His6 containing the TATA box and transcription start sites was introduced at the 5′ end of mLm-SV40. The 443 bp Dmn promoter, amplified from genomic DNA with primers Dmn_Pro_Xho1 (GCCTCGAGCACGAAAACTACAGTGTTGAC) and Dmn_Pro2_Nco1 (CTCCATGGTTACCAAACAAATGCCAAAGTGCAG), was inserted into this plasmid by Xho 1 and Nco 1 digestion. A Xho 1 fragment containing the Dmn promoter, proximal regulatory elements and mLm-SV40 was then moved into an existing pUASTB-based plasmid (Groth et al. 2004) containing *roX1*, 1.688^3F^ repeats and w ^+ mC^ (Joshi and Meller 2017) that had been modified by conversion of Mlu 1 flanking the attP site into Xho 1. *roX1* is contained in a 4.9 kb EcoR1 fragment (Kelley et al. 1999) and 1.688^3F^ repeats are contained within a 2 kb amplicon generated with flanking primers (CGGGATCCCCCACCAAGAGGCTTGACAGAAGA and TCCCCGCGGGTGGCGAAAGGTTATGGAGATGACC) (Menon et al. 2014) in a reaction templated with DNA from our laboratory *yw* strain, which carries more repeats at this location than the reference scaffold. Construction was verified by restriction mapping, sequencing, and PCR at each stage. A diagram of the final construct is presented in Supplementary Fig. 1. Integration at PBac{y[ + ]-attP-3B}VK00033 (65B2) was performed by Rainbow Transgenics (Camarillo, CA) and verified by single fly PCR. Matings scheme that generated RNAi knockdown in the different constructs are presented in Supplementary Fig. 2. FlyBase (release 6.56) was used to access information on promoter activity and sequence (Arzu Öztürk-Çolak et al. 2024).

### Embryo and adult lysate preparation

Six to twelve hour embryos were collected on molasses agar plates smeared with yeast paste. Collection time is a developmental window where the promoters driving Fluc and Rluc expression display minimal change. Embryos were washed with embryo wash buffer (0.03% Triton X-100, 0.04% NaCl), dechorionated with 50% bleach solution for 2 min and rinsed with tap water. Dechorionated embryos were homogenized in five volumes lysis buffer (1% Triton X-100, 10% glycerol, 25 mM glycylglycine pH 7.8, 15 mM MgSO_4_, 4 mM EGTA, 1 mM DTT). Lysate was centrifuged for 5 min, 13,000 RPM at 4°C to remove debris. Aliquots of supernatant were frozen at −80°C. One day old flies were frozen at −20°C for at least 30 min before squashing in lysis buffer (100 μL/fly) and centrifuged to remove debris. Aliquots of supernatant were frozen at −20°C.

### Firefly luciferase and dual luciferase assays

Duplicate assays of 20 μL of lysate were analyzed in a 96-well plate using a protocol adapted from Dyer et al. (2000). One hundred μL of Firefly luciferase assay buffer (25 mM glycylglycine [Fisher, cat # AC120140250], 15 mM KxPO4 [mixture of monobasic dihydrogen phosphate and dibasic monohydrogen phosphate, pH 8.0], 4 mM EGTA, 2 mM ATP, 1 mM DTT, 15 mM MgSO_4_, 0.1 mM Coenzyme A [Nanolight, #309], 75 μM luciferin [Nanolight, #306], pH 8.0) was added and luminescence was measured two seconds after buffer addition in an endpoint read at all wavelengths. This was followed by addition of 100 μL of Renilla assay buffer (1.1 M NaCl, 2.2 mM EDTA, 0.22 M KxPO4 at pH of 5.1, 0.44 mg/mL BSA, 2 mM NaN_3_, 10 μM coelenterazine [Nanolight, #303], pH adjusted to 5.0). Luminescence was measured two seconds after buffer addition using Spectramax i3x (Molecular Devices) or a GloMax microplate luminometer (Promega).

### Quantitative real time PCR

Sixty third instar wandering larvae or 100 μL of 0–12 h embryos were homogenized in Trizol reagent (Invitrogen) and RNA isolated according to manufacturer instructions. Total RNA was cleaned using the Qiagen RNeasy Mini kit. One microgram of total RNA was reverse transcribed at 42°C for 1 h using ImProm-IV reverse transcriptase (Promega) and the cDNA template amplified using iTaqUniversal SYBR Green Supermix (Bio-Rad). Expression was normalized to *Dmn* (DCTN2-p50). The level of mRNA knock down appears modest in most cases (Supplementary Fig. 3). However, the anticipated phenotypes are observed (complete lethality upon *CLAMP* knock down and male-specific *msl2* lethality). We conclude that partial knock down is sufficient to investigate the genes of interest. Activity of the *spaghetti squash* (*sqh*) promoter used in these studies is similar in 0–12 h embryos, wandering third instar larvae and adults as determined by mRNA sequencing (Graveley et al. 2011).

## Results

### Reporter system design

**Fig. 1.**
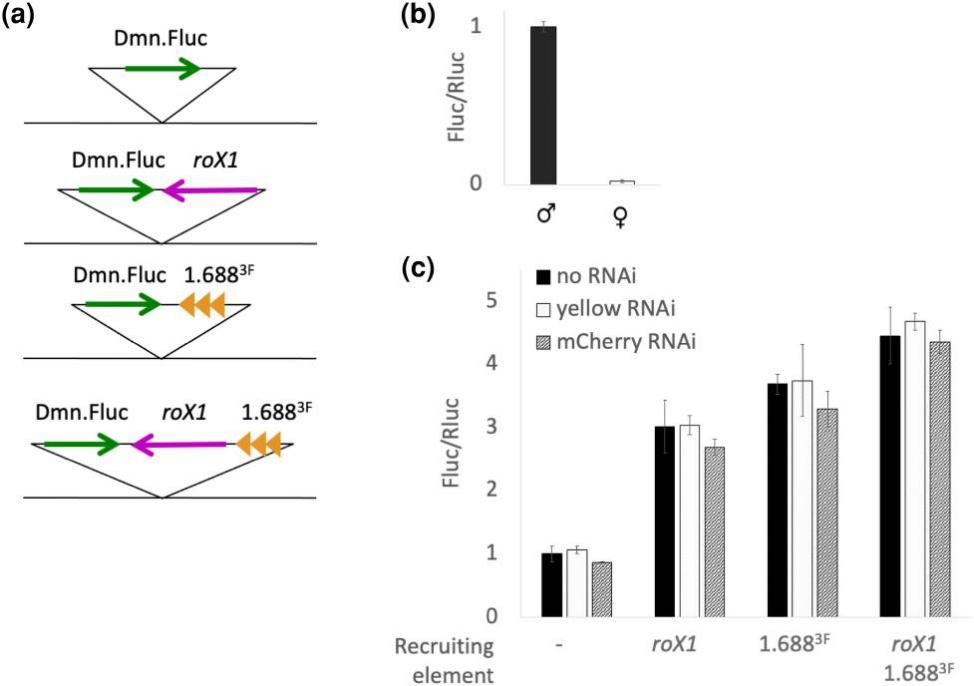
Reporter system to measure recruitment of dosage compensation. a) Firefly luciferase reporters driven by the *Dmn* promoter. Recruiting elements *roX1* and 1.688^X^ are introduced nearby. b) Luciferase activity is easily detected in lysates from male flies. Female flies show no activity. Fluc is normalized to *Renilla* luciferase (Rluc) driven by *P*{sqh-GAL4}2. Error bars represent SEM of three biological replicates. c) Luciferase activity increases in the presence of recruiting elements (black). Knockdown of *yellow* (hatched gray) or mCherry (hatched black) does not affect activity. Values represent Fluc activity normalized to Rluc activity and divided by the Fluc/Rluc ratio of the control with no recruiting element. No knock down controls are generated by mating males with the reporter to a *yw* laboratory reference strain. Error bars represent SEM of three biological replicates.

Recruiting elements, either *roX1*, 1.688^X^ DNA, or both, next to a Firefly luciferase reporter (Fluc) were integrated at 65B2 on the third chromosome (Fig. 1a). Both *roX* genes contain internal CES and are able to recruit compensation to nearby genes when inserted on an autosome (Kelley et al. 1999; Henry et al. 2001; Park et al. 2003). We have previously evaluated three 1.688^X^ repeats, 1.688^1A^, 1.688^3C^, and 1.688^3F^ (superscript designates cytological location on the X), for ability to attract compensation to an autosomal insertion site and found that all recruit to a similar level (Joshi and Meller 2017). 1.688^3F^ serves as our laboratory reference and is used in these studies. *Renilla* luciferase on the second chromosome serves as the internal control (Rluc). Fluc is engineered to be expressed only in males, the sex in which dosage compensation occurs. In brief, the *msl2* 5′ and 3′ UTRs, which contain SXL (Sexlethal) binding sites, flank the Fluc coding region (described in Gebauer et al. (1999)). SXL, which is expressed only in females, binds the mRNA to block translation (Kelley et al. 1997). This limits Fluc expression to males, enabling screening of essential genes in mixed sex embryos. Recruiting elements are flanked by Flp or Cre sites to enable excision from the transgene, allowing testing of individual elements. Controls with no recruiting elements were also generated (Supplementary Fig. 1). Lysates of male and female adults carrying Fluc driven by the Dynactin 2, p50 subunit (*Dmn*) promoter were assayed for luciferase activity. Males showed strong luciferase activity, but females had little to no luciferase activity (Fig. 1b). We conclude that the Firefly luciferase reporter is functional, and that expression is blocked in females by the SXL binding sites.

We then tested adult male lysates for Firefly luciferase expression in the presence of recruiting elements using the mating scheme shown in Supplementary Fig. 2. The presence of both *roX1* and 1.688^X^ elevates luciferase expression ∼4-fold in comparison to Fluc only control, and single recruiting elements (either *roX1* or 1.688^3F^ repeats) show a 3–3.5-fold increase in expression (Fig. 1c, black bars). As a control for RNAi induction, and for the presence of an additional UAS site, we measured luciferase expression in adult males knocked down for *yellow* (*y*) to engage RNAi with an endogenous target. We also performed RNAi knock down of mCherry, which lacks a target (Fig. 1c). Both control knock downs produced Fluc/Rluc ratios essentially identical to those from mating to our lab reference *yellow white* (*yw*) strain, revealing that neither an additional UAS site nor engagement of the RNAi system influences the reporter.

### Reporter response differs in embryos and adults

**Fig. 2.**
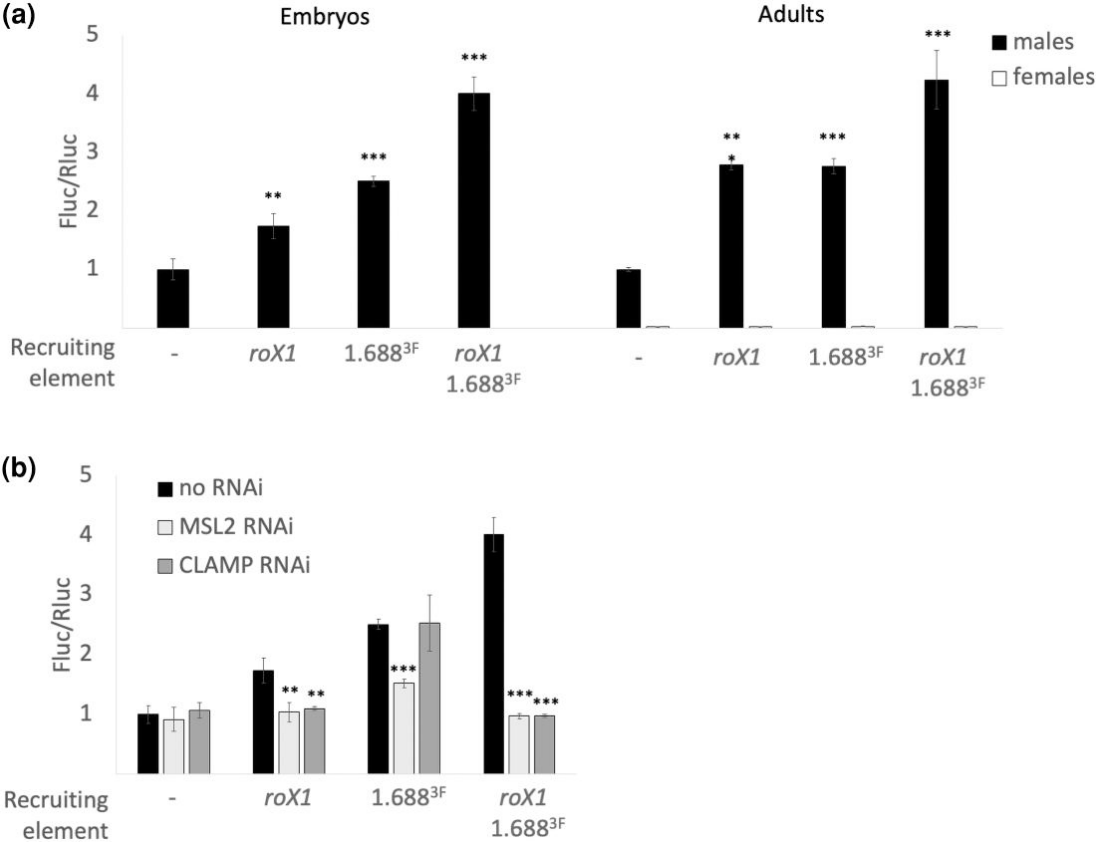
Reporter validation by knock down of dosage compensation genes. a) *Dmn*.Fluc response to recruiting elements in embryos and adults. Left: Activity in embryos. *Dmn*.Fluc with *roX1* or 1.688^X^ shows 1.7-fold and 2.5-fold increase in activity, respectively, compared to no recruiting element. Activity is elevated almost 4-fold when both *roX1* and 1.688^X^ are present. Right: *Dmn*.Fluc in adult flies. Females show no Fluc activity (white). Activity in lysates from male flies (black) increases almost 3-fold in the presence of single recruiting elements and 4-fold with both elements. Significance is relative to the no recruiting element control. b) *Dmn*.Fluc activity in mixed sex embryos with recruiting elements decreases upon MSL2 knock down (light gray) compared to control (black). CLAMP knock down (dark gray) decreases Fluc expression when a *roX1* recruiting element is present but recruitment by 1.688^X^ alone is unaffected. Significance is relative to no RNAi control. Fluc is normalized to Rluc and divided by the Fluc/Rluc ratio of no recruiting element control. Error bars represent SEM of three biological replicates. Significance was determined using Student's two sample t-test (*P ≤ 0.01, **P ≤ 0.001). RNAi lines are described in Supplementary Table 1.

We then compared Fluc activity in mixed-sex embryos and sexed adults (Fig. 2a). As expected, lysates from adult males showed strong luciferase activity for all constructs, but females displayed no activity (Fig. 2a, right). The response to recruiting elements was similar in embryos and adults, but stage-specific differences were noted. Inclusion of *roX1* and 1.688^X^ elevated Fluc expression 4-fold in embryos and adult males. *roX1* increases Fluc expression by 1.7-fold in embryos (Fig. 2a, left), compared to a 2.8-fold increase in adults (Fig. 2a, right). Lastly, 1.688^X^ repeats alone elevate expression to similar levels in both life stages, 2.5-fold in embryos and 2.8-fold in adults (Fig. 2a, right). The difference in activation by 1.688^X^ repeats and *roX1* in embryos is significant, as is the difference in activation by *roX1* alone in embryos and adults (*P*-values of 0.007 and 0.003, respectively). This suggests that 1.688^X^ repeats play a more prominent role in X recognition in embryos, a time when compensation is being established. In contrast, the contributions of 1.688^X^ repeats and *roX1* are more equivalent later in development. In spite of this difference, when both elements are present a 4-fold activation is observed in both life stages. This is much higher than the 2-fold increase anticipated by compensation alone. Previous studies have demonstrated that recruitment of the MSL complex by autosomal *roX1* transgenes overcomes local chromatin silencing in males (Kelley and Kuroda 2003). Activation of the Fluc reporter thus represents the combined effects of removal of repression and activation by MSL complex activity. The degree of Fluc activation would consequently vary depending on chromatin environment at the integration site, but it is important to note that both effects rely on recruitment of the MSL complex, precisely what we seek to measure.

### Validation by knock down of genes known to participate in dosage compensation

We first determined the effect of *msl2* knock down in mixed sex embryos, a treatment expected to block all activation due to recruitment of compensation, using the ubiquitous *sqh*-GAL4 driver. As a control, flies were mated to our lab reference *yw* strain (no RNAi; black bars Fig. 2b). As expected, *msl2* knock down reduced Fluc activation by recruiting elements but had no effect on Fluc alone (Fig. 2b, light gray). To determine if the embryo population was skewed to females by *msl2* knock down, we performed X-Gal staining of embryos that express *LacZ* in females and are knocked down for *msl2,* and found similar sex ratios in 6–12 h embryo collections with and without *msl2* knock down (Supplementary Fig. 4; Estes et al. 1995). Next, we tested recruitment in embryos knocked down for *CLAMP*. Because *CLAMP* binds the CES in *roX1*, we expected knock down to block activation only when *roX1* is present. CLAMP knockdown is lethal to both sexes, limiting this study to embryos. Knocking down CLAMP reduces Fluc activity in transgenes with *roX1* and in transgenes with both recruiting elements but does not affect recruitment by 1.688^X^ alone or when no recruiting element is present (Fig. 2b, dark gray). This supports the idea that CLAMP operates at CES, and that CES and 1.688^X^ repeats recruit in a mechanistically distinct way.

### Response to knock down of small RNA pathway components is complex

**Fig. 3.**
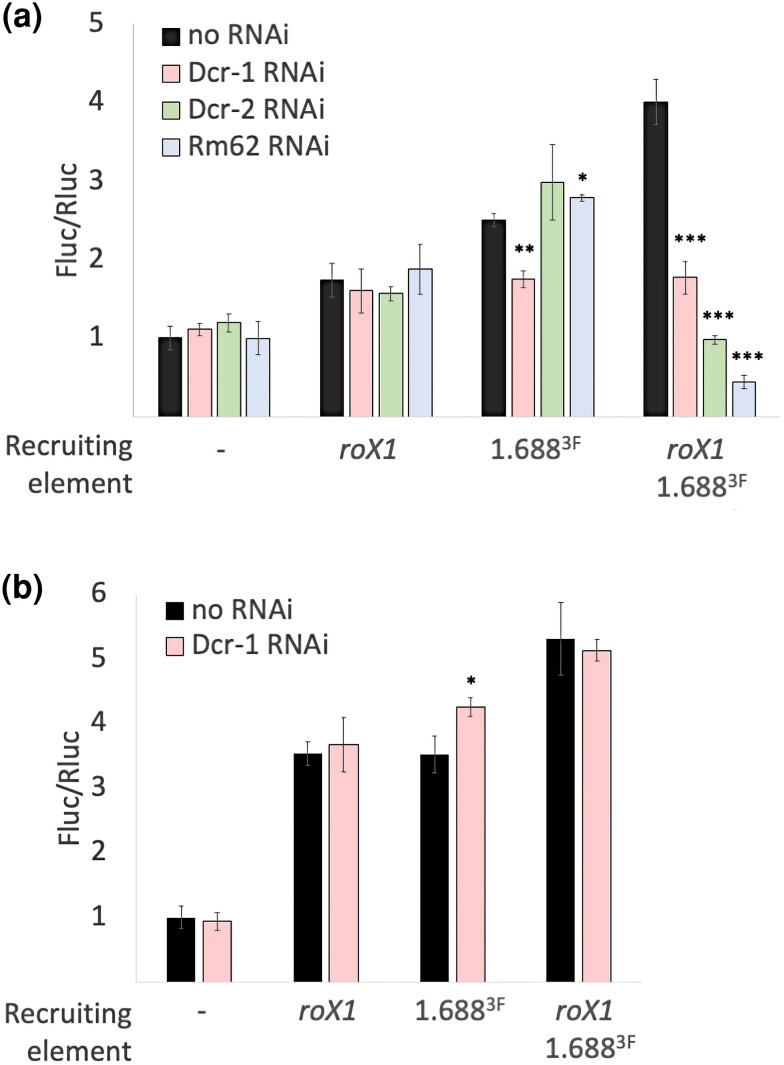
Knock downs in small RNA pathways reveal complexity in recruitment. a) *Dmn*.Fluc activity in embryos. Dcr-1 knock down (pink) selectively reduces Fluc with 1.688^X^ or *roX1* and 1.688^X^ but is unaffected when *roX1* alone is present. *Dmn*.Fluc activity decreases upon Dcr-2 (green) or Rm62 (blue) knock down when both *roX1* and 1.688^X^ are present but is unaffected in reporters with single recruiting elements. b) *Dmn*.Fluc activity is unaffected when Dcr-1 is knocked down in adult males. Significance is relative to no RNAi controls generated by mating flies with reporter to our lab reference *yw* strain. Fluc is normalized to Rluc and the ratio of each sample divided by the Fluc/Rluc ratio of the control without recruiting element. Error bars represent SEM of three biological replicates. Significance was determined using Student's two sample t-test (*P ≤ 0.01, **P ≤ 0.001). RNAi lines are described in Supplementary Table 1.

Mutations in many genes involved in small RNA production or action enhance the male lethality of *roX1 roX2* mutations, suggesting that small RNA contributes to X recognition (Deshpande and Meller 2018). We focused our analysis on Dicer-1 (Dcr-1), Dicer-2 (Dcr-2), and Rm62, genes that, when mutated, act as dominant enhancers of *roX1 roX2* male lethality (Deshpande and Meller 2018). Dcr-1 and Dcr-2 process dsRNA into siRNA and play a role in sorting small RNAs to effector complexes (Lee et al. 2004, Lim et al. 2008). Rm62 is an RNA helicase that is involved in splicing and small RNA pathways (Ishizuka et al. 2002). All three proteins participate in formation of silent chromatin and interact with Argonaute 2 (Ago2). We propose that small RNA from 1.688^X^ repeats enhances X recognition through modification of chromatin at cognate elements on the X and anticipated that knock down would affect recruitment by the 1.688^X^ element but not *roX1*. In accord with this, knocking down Dcr-1 in embryos reduces Fluc activity from transgenes with 1.688^X^ repeats or with *roX1* and 1.688^X^, but does not affect recruitment by *roX1* alone (Fig. 3a, pink). In comparison, we see no reduction in Fluc activity in adults (Fig. 3b). This suggests a more critical role for Dcr-1 in recruitment through 1.688^X^ repeats early in development and supports the idea that 1.688^X^ repeats play a role at this stage. As knockdown of Dcr-2 and Rm62 is lethal to both sexes, these were examined only in embryos. Unexpectedly, recruitment by single elements was unaffected by knocking down either Dcr-2 (Fig. 3a, green) or Rm62 (Fig. 3a, blue). Instead, we see a dramatic reduction in Fluc activity from transgenes with both *roX1* and 1.688^X^ elements. This observation is hard to explain but striking in that both genes participate in small RNA pathways, modulate chromatin, and interact with shared proteins. We speculate that knock down of Rm62 and Dcr-2 neutralizes the ability of these elements to recruit only when both are present. Why this would occur is unclear. We expect that knockdown is impacting multiple pathways in a complex manner.

### Evaluating small RNA effectors

**Fig. 4.**
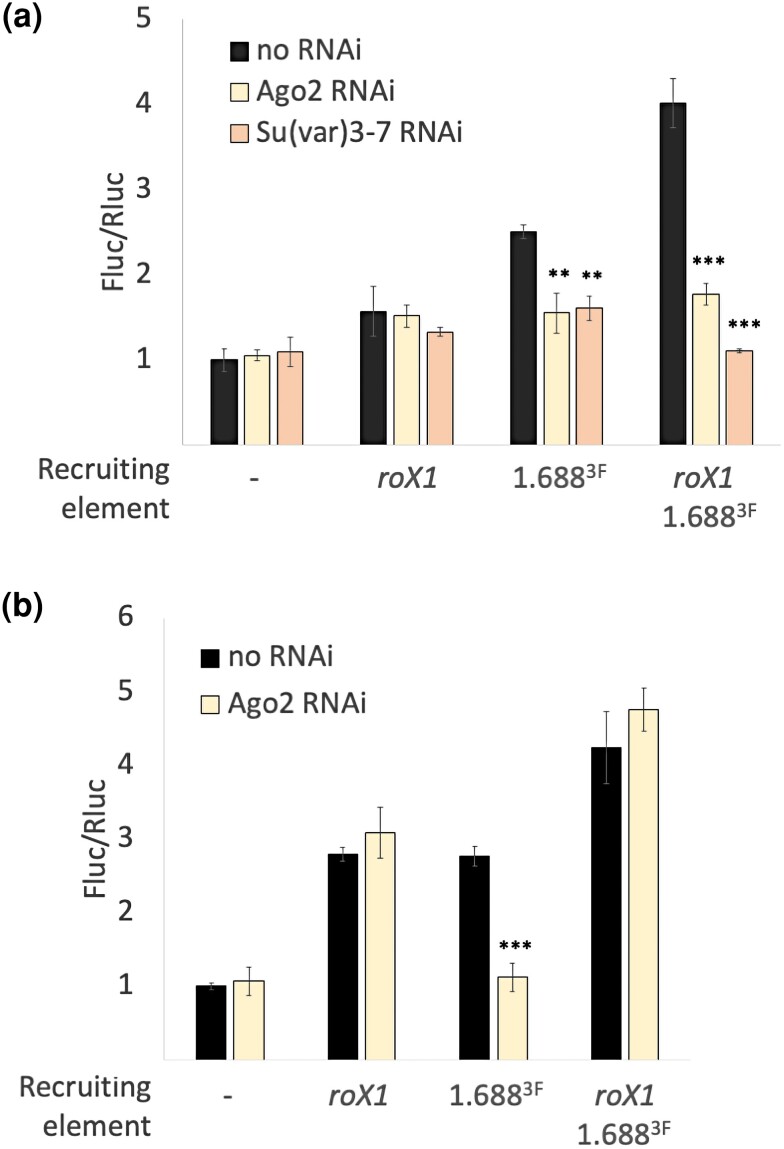
Knock down of small RNA effectors impairs recruitment by 1.688^X^. a) *Dmn*.Fluc activity in embryos decreases when Ago2 (yellow) or Su(var)3-7 (orange) is knocked down in reporter with 1.688^X^, or *roX1* and 1.688^X^, but is unaffected in reporter with *roX1* alone. b) *Dmn*.Fluc activity in adult males decreases when Ago2 (yellow) is knocked down in reporter with 1.688^X^, but not in reporters with *roX1* or *roX1* and 1.688^X^. Significance is relative to no RNAi control generated by mating flies with reporter to our laboratory reference *yw* strain. Fluc is normalized to Rluc and the Fluc/Rluc ratio of each sample is divided by the Fluc/Rluc ratio of the no recruiting element control. Error bars represent SEM of three biological replicates. Significance was determined using Student's two sample t-test (*P ≤ 0.01, **P ≤ 0.001). RNAi lines are described in Supplementary Table 1.

Previous studies showed that reduction of the effector protein Argonaute2 (Ago2), and of several Ago2-interacting proteins including the H3K9 methyltransferase Su(var)3–9, reduces survival of *roX1 roX2* mutant males (Menon and Meller 2012; Deshpande and Meller 2018). Su(var)3–9 is responsible for enrichment of H3K9me2 at many 1.688^X^ repeats. In addition, Ago2 localizes at 1.688^X^ repeats (Deshpande and Meller 2018). This suggests that an Ago2-containing chromatin modifier complex acts at 1.688^X^ repeats to help identify the X. Ago2 knock down reduces Fluc activity in embryos and adults with 1.688^X^ recruiting elements (Fig. 4, a and b, yellow). In contrast, Ago2 knockdown does not affect recruitment by *roX1* alone. We also detect a reduction in embryonic Fluc activity when both recruiting elements are present (Fig. 4a, yellow), but this is not seen in adults (Fig. 4b, yellow).

Su(var)3–9 is of great interest as it places H3K9me2 at some 1.688^X^ repeats, interacts with Ago2 and mutations in Su(var)3–9 enhance *roX1 roX2* male lethality. However, available Su(var)3–9 RNAi lines also target an essential overlapping gene, the initiation factor eIF2γ. We tested flies heterozygous for a Su(var)3–9 mutation previously shown to enhance *roX1 roX2* male lethality and saw no effect on Fluc activity (Supplementary Fig. 5). We then tested knock down of Su(var)3–7, which interacts with Su(var)3–9 (Schotta et al. 2002). Loss of a single copy of Su(var)3–7 enhances *roX1 roX2* male lethality (Deshpande and Meller 2018). Knocking down Su(var)3–7 reduces Fluc activity in transgenes with 1.688^X^ repeats or with *roX1* and 1.688^X^ (Fig. 4a, orange). In comparison, Su(var)3–7 knock down does not affect recruitment by *roX1* alone. We propose that Su(var)3–7 is a component of a chromatin modification system that functions in recruitment by 1.688^X^, but not *roX1*.

## Discussion

Although the MSL complex has been intensively studied, selective targeting of this complex to the X chromosome is poorly understood. A two-step model has been proposed in which CLAMP binds to GA-rich motifs in CES on the X chromosome and recruits the MSL complex. This is followed by spreading to nearby active genes through the interaction of MSL3 with the cotranscriptional H3K36me3 mark. In accord with this idea, highly expressed genes that are more enriched for H3K36me3 are most fully compensated (Alekseyenko et al. 2006; Joshi and Meller 2017). This model for X recognition is derived from genome-wide studies of MSL protein, *roX* RNA or CLAMP binding, and reflects endpoint patterns of localization (Alekseyenko et al. 2006, Gilfillan et al. 2006, Legube et al. 2006, Kind et al. 2008, Straub et al. 2008, Larschan et al. 2011, Ferrari et al. 2013; Soruco et al. 2013). Our understanding of the nature of recruiting elements, as well as their molecular functions and interactions, is incomplete in this context. To address this, we designed a reporter system capable of dissecting the genetic requirements for recruitment by different DNA elements. Unlike a previous functional test of recruitment that relied on complex genotypes to demonstrate autosomal compensation, our system accommodates screening of candidate genes by RNAi knock down (Joshi and Meller 2017). It could also be adapted to quantitatively measure recruitment by any *cis*-acting element. Validation by knock down of MSL2 confirmed recruitment of compensation by CES-containing *roX1* and 1.688^X^ repeats. CLAMP, which binds the CES, was found to operate solely through *roX1* and had no effect when only 1.688^X^ repeats were present. This confirms that the CES and 1.688^X^ repeats both recruit the MSL complex but operate through different pathways.

A caveat to this approach is that elements within genes and promoters that contribute to compensation are poorly defined and capable of interference. A few promoters are known to recruit compensation, the best known of these is the X-linked *white (w)* gene. Most transgenes are marked with *w*, and expression of this marker is usually compensated when placed on an autosome (Pirrotta et al. 1985; Laverty et al. 2011). The Fluc constructs carrying *roX1* alone and both recruiting elements carry “miniwhite” (*w^+ mC^*; see Supplementary Fig. 1), and all are inserted at a landing site marked with *y^+^*, another X-linked and compensated gene (Karchenko et al. 2011). In a previous study, we found no influence of an insertion marked with *y^+^* and *w^+ mC^* on expression of a nearby gene, suggesting minimal effect (Joshi and Meller 2017). Furthermore, response to knock down of genes that operate through single recruiting elements, such as CLAMP, Ago2 and Su(var)3–7, effectively eliminates Fluc activation when a sole recruiting element is present. This suggests that recruitment by transgene-associated markers is unlikely to influence conclusions.

The signature defect in *roX1 roX2* males is reduced X recognition. Many factors have been implicated in dosage compensation by screening for enhancement of *roX1 roX2* male lethality (Menon and Meller 2012; Deshpande and Meller 2018). We postulate that some of these genes are likely to participate in recruitment of compensation, but this test fails to identify the recruiting element that is involved. Based on previous studies, we anticipated that knock downs affecting the siRNA pathway would block recruitment by 1.688^X^ repeats. As expected, knock down of Dcr-1 reveals a role in establishment of compensation in embryos through the 1.688^X^ repeats. In contrast, knock down of Rm62 or Dcr-2 had no effect on individual recruiting elements but fully neutralized recruitment when both were present together, an observation that we cannot fully explain. This highlights the potential complexity of interactions between recruiting elements. In light of this, it is interesting that the CES in *roX1* recruits boundary elements containing factors that intersect with small RNA pathways (Kaye et al. 2017). It is possible that the close proximity of *roX1* and 1.688^X^ elements in our transgene enables repression in a specific context.

Ago2 binds to some 1.688^X^ repeats, and loss of Ago2 in a partial loss of function *roX1 roX2* background is male lethal and accompanied by loss of MSL binding to the X chromosome (Menon and Meller 2012; Deshpande and Meller 2018). We now confirm a role for Ago2 in recruitment by 1.688^X^ repeats. Knock down of Su(var)3–7, an interactor of Su(var)3–9, also operates through 1.688^X^ repeats. Su(var)3–7 is necessary for heterochromatin formation and silencing, functions that are dependent on Su(var)3–9 activity (Spierer et al. 2005, 2008). Interestingly, both genes have been linked to the compensated male X chromosome. Reduction of Su(var)3–9 and reduction or overexpression of Su(var)3–7 disrupt the morphology of the polytenized male X chromosome, an effect that is dependent on an active MSL complex (Spierer et al 2005). It counterintuitive that an epigenetic silencing system promotes compensation. We postulate that enrichment of H3K9me2 helps mark the X for compensation. It has been noted that the male X is modestly enriched for HP1, a heterochromatin protein that binds the H3K9me2 mark placed by Su(var)3–9 (de Wit et al. 2005). Furthermore, increased deposition of H3K9me2 around an autosomal 1.688^X^ insertion is associated with increased expression of nearby genes (Deshpande and Meller 2018). Our findings suggest that recruitment of Ago2 and placement of repressive marks is integral to identification of X chromatin for dosage compensation.

In addition to differentiating the mechanism of recruitment by 1.688^X^ and CES, this study reveals that recruiting elements act collaboratively in embryos and adults but suggests that 1.688^X^ repeats play a larger role during embryogenesis. This may reflect the mechanism of recruitment by each element. The CES are involved in long-range interactions that facilitate spreading of the MSL complex (Grimaud and Becker 2009; Ramírez et al. 2015). It is possible that these interactions are disrupted by rapid cycles of replication in embryos. In contrast, the nonreplicating cells of adults may have stable long-range interactions that favor activation by CES. The relative contribution of the 1.688^X^ repeats is higher in embryos, suggesting that satellite repeats drive X identification in early development. Indeed, maternally provided repeat-associated small RNAs are detected in early embryos, but are less abundant at later developmental stages, and may play a role in initial X recognition (Aravin et al. 2003; Menon et al. 2014). These differences suggest a temporal shift in recruitment of compensation during development. Both elements, however, work together and highest compensation is observed when both are present regardless of life stage. Nevertheless, our findings suggest that models put forth for the establishment of compensation are incomplete.

## Supplementary Material

jkae057_Supplementary_Data

## Data Availability

Details of transgene construction are available in Materials and Methods, Supplementary Material and from the authors. Fly strains may be obtained from the Bloomington Drosophila Stock Center, the Vienna Stock Center, or by contacting the authors. The authors affirm that all data necessary for confirming the conclusions of the article are present within the article, figures, and tables. Supplemental material available at G3 online.

## References

[jkae057-B1] Akhtar A, Becker PB. 2000. Activation of transcription through histone H4 acetylation by MOF, an acetyltransferase essential for dosage compensation in Drosophila. Mol Cell. 5(2):367–375. doi:10.1016/s1097-2765(00)80431-1. 10882077

[jkae057-B2] Alekseyenko AA, Larschan E, Lai WR, Park PJ, Kuroda MI. 2006. High-resolution ChIP-chip analysis reveals that the Drosophila MSL complex selectively identifies active genes on the male X chromosome. Genes Dev. 20(7):848–857. doi:10.1101/gad.1400206. 16547173 PMC1472287

[jkae057-B3] Alekseyenko AA, Peng S, Larschan E, Gorchakov AA, Lee OK, Kharchenko P, McGrath SD, Wang CI, Mardis ER, Park PJ, et al 2008. A sequence motif within chromatin entry sites directs MSL establishment on the Drosophila X chromosome. Cell. 134(4):599–609. doi:10.1016/j.cell.2008.06.033. 18724933 PMC2613042

[jkae057-B4] Aravin AA, Lagos-Quintana M, Yalcin A, Zavolan M, Marks D, Snyder B, Gaasterland T, Meyer J, Tuschl T. 2003. The small RNA profile during Drosophila melanogaster development. Dev Cell. 5(2):337–350. doi:10.1016/s1534-5807(03)00228-4. 12919683

[jkae057-B5] Bell O, Conrad T, Kind J, Wirbelauer C, Akhtar A, Schübeler D. 2008. Transcription-coupled methylation of histone H3 at lysine 36 regulates dosage compensation by enhancing recruitment of the MSL complex in Drosophila melanogaster. Mol Cell Biol. 28(10):3401–3409. doi:10.1128/MCB.00006-08. 18347056 PMC2423169

[jkae057-B6] Brockdorff N, Turner BM. 2015. Dosage compensation in mammals. Cold Spring Harb Perspect Biol. 7(3):a019406. doi:10.1101/cshperspect.a019406. 25731764 PMC4355265

[jkae057-B7] Deshpande N, Meller VH. 2018. Chromatin that guides dosage compensation is modulated by the siRNA pathway in *Drosophila melanogaster*. Genetics. 209(4):1085–1097. doi:10.1534/genetics.118.301173. 29921620 PMC6063223

[jkae057-B8] de Wit E, Greil F, van Steensel B. 2005. Genome-wide HP1 binding in Drosophila: developmental plasticity and genomic targeting signals. Genome Res. 15(9):1265–1273. doi:10.1101/gr.3198905. 16109969 PMC1199541

[jkae057-B9] DiBartolomeis SM, Tartof KD, Jackson FR. 1992. A superfamily of Drosophila satellite related (SR) DNA repeats restricted to the X chromosome euchromatin. Nucleic Acids Res. 20(5):1113–1116. doi:10.1093/nar/20.5.1113. 1549474 PMC312099

[jkae057-B10] Disteche CM . 2012. Dosage compensation of the sex chromosomes. Annu Rev Genet. 46(1):537–560. doi:10.1146/annurev-genet-110711-155454. 22974302 PMC3767307

[jkae057-B11] Dyer BW, Ferrer FA, Klinedinst DK, Rodriguez R. 2000. A noncommercial dual luciferase enzyme assay system for reporter gene analysis. Anal Biochem. 282(1):158–161. doi:10.1006/abio.2000.4605. 10860516

[jkae057-B12] Estes PA, Keyes LN, Schedl P. 1995. Multiple response elements in the sex-lethal early promoter ensure its female-specific expression pattern. Mol Cell Biol. 15(2):904–917. doi:10.1128/MCB.15.2.904. 7823955 PMC231975

[jkae057-B13] Ferrari F, Plachetka A, Alekseyenko AA, Jung YL, Ozsolak F, Kharchenko PV, Park PJ, Kuroda MI. 2013. Jump start and gain” model for dosage compensation in Drosophila based on direct sequencing of nascent transcripts. Cell Rep. 5(3):629–636. doi:10.1016/j.celrep.2013.09.037. 24183666 PMC3852897

[jkae057-B14] Gebauer F, Corona DF, Preiss T, Becker PB, Hentze MW. 1999. Translational control of dosage compensation in Drosophila by sex-lethal: cooperative silencing via the 5′ and 3′ UTRs of msl-2 mRNA is independent of the poly(A) tail. EMBO J. 18(21):6146–6154. doi:10.1093/emboj/18.21.6146. 10545124 PMC1171678

[jkae057-B15] Gelbart ME, Kuroda MI. 2009. Drosophila dosage compensation: a complex voyage to the X chromosome. Development (Cambridge, England). 136(9):1399–1410. doi:10.1242/dev.029645. 19363150 PMC2674252

[jkae057-B16] Gilfillan GD, Straub T, de Wit E, Greil F, Lamm R, van Steensel B, Becker PB. 2006. Chromosome-wide gene-specific targeting of the Drosophila dosage compensation complex. Genes Dev. 20(7):858–870. doi:10.1101/gad.1399406. 16547172 PMC1475731

[jkae057-B17] Graveley B, Brooks A, Carlson J, Duff MO, Landolin JM, Yang L, Artieri CG, van Baren MJ, Boley N, Booth BW, et al 2011. The developmental transcriptome of Drosophila melanogaster. Nature. 471(7339):473–479. doi:10.1038/nature09715. 21179090 PMC3075879

[jkae057-B18] Grimaud C, Becker PB. 2009. The dosage compensation complex shapes the conformation of the X chromosome in Drosophila. Genes Dev. 23(21):2490–2495. doi:10.1101/gad.539509. 19884256 PMC2779748

[jkae057-B19] Groth AC, Fish M, Nusse R, Calos MP. 2004. Construction of transgenic Drosophila by using the site-specific integrase from phage ΦC31. Genetics. 166(4):1775–1782. doi:10.1093/genetics/166.4.1775. 15126397 PMC1470814

[jkae057-B20] Henry RA, Tews B, Li X, Scott MJ. 2001. Recruitment of the male-specific lethal (MSL) dosage compensation complex to an autosomally integrated roX chromatin entry site correlates with an increased expression of an adjacent reporter gene in male Drosophila. J Biol Chem. 276(34):31953–31958. doi:10.1074/jbc.M103008200. 11402038

[jkae057-B21] Ilik IA, Quinn JJ, Georgiev P, Tavares-Cadete F, Maticzka D, Toscano S, Wan Y, Spitale RC, Luscombe N, Backofen R, et al 2013. Tandem stem-loops in roX RNAs act together to mediate X chromosome dosage compensation in Drosophila. Mol Cell. 51(2):156–173. doi:10.1016/j.molcel.2013.07.001. 23870142 PMC3804161

[jkae057-B22] Ishizuka A, Siomi MC, Siomi H. 2002. A Drosophila fragile X protein interacts with components of RNAi and ribosomal proteins. Genes Dev. 16(19):2497–2508. doi:10.1101/gad.1022002. 12368261 PMC187455

[jkae057-B23] Joshi SS, Meller VH. 2017. Satellite repeats identify X chromatin for dosage compensation in Drosophila melanogaster males. Curr Biol. 27(10):1393–1402.e2. doi:10.1016/j.cub.2017.03.078. 28457869 PMC5497753

[jkae057-B901] Kharchenko P, Alekseyenko A, Schwartz Y, Minoda A, Riddle NC, Ernst J, Sabo PJ, Larschan E, Gorchakov AA, Gu T , et al 2011. Comprehensive analysis of the chromatin landscape in Drosophila melanogaster. Nature. 471, 480–485. doi:10.1038/nature09725. PMC3109908 21179089

[jkae057-B24] Kaye EG, Kurbidaeva A, Wolle D, Aoki T, Schedl P, Larschan E. 2017. Drosophila dosage compensation loci associate with a boundary-forming insulator Complex. Mol Cell Biol. 37(21):e00253-17. doi:10.1128/MCB.00253-17. PMC5640815 28784719

[jkae057-B25] Kelley RL, Kuroda MI. 2003. The Drosophila roX1 RNA gene can overcome silent chromatin by recruiting the male-specific lethal dosage compensation complex. Genetics. 164(2):565–574. doi:10.1093/genetics/164.2.565. 12807777 PMC1462573

[jkae057-B26] Kelley RL, Meller VH, Gordadze PR, Roman G, Davis RL, Kuroda MI. 1999. Epigenetic spreading of the Drosophila dosage compensation complex from roX RNA genes into flanking chromatin. Cell. 98(4):513–522. doi:10.1016/s0092-8674(00)81979-0. 10481915

[jkae057-B27] Kelley RL, Wang J, Bell L, Kuroda MI. 1997. Sex lethal controls dosage compensation in Drosophila by a non-splicing mechanism. Nature. 387(6629):195–199. doi:10.1038/387195a0. 9144292

[jkae057-B28] Kind J, Vaquerizas JM, Gebhardt P, Gentzel M, Luscombe NM, Bertone P, Akhtar A. 2008. Genome-wide analysis reveals MOF as a key regulator of dosage compensation and gene expression in Drosophila. Cell. 133(5):813–828. doi:10.1016/j.cell.2008.04.036. 18510926

[jkae057-B29] Kuhn GC, Küttler H, Moreira-Filho O, Heslop-Harrison JS. 2012. The 1.688 repetitive DNA of Drosophila: concerted evolution at different genomic scales and association with genes. Mol Biol Evol. 29(1):7–11. doi:10.1093/molbev/msr173. 21712468

[jkae057-B30] Larschan E, Alekseyenko AA, Gortchakov AA, Peng S, Li B, Yang P, Workman JL, Park PJ, Kuroda MI. 2007. MSL complex is attracted to genes marked by H3K36 trimethylation using a sequence-independent mechanism. Mol Cell. 28(1):121–133. doi:10.1016/j.molcel.2007.08.011. 17936709

[jkae057-B31] Larschan E, Bishop EP, Kharchenko PV, Core LJ, Lis JT, Park PJ, Kuroda MI. 2011. X chromosome dosage compensation via enhanced transcriptional elongation in Drosophila. Nature. 471(7336):115–118. doi:10.1038/nature09757. 21368835 PMC3076316

[jkae057-B32] Laverty C, Li F, Belikoff EJ, Scott MJ. 2011. Abnormal dosage compensation of reporter genes driven by the Drosophila glass multiple reporter (GMR) enhancer-promoter. PloS one. 6(5):e20455. doi:10.1371/journal.pone.0020455. 21655213 PMC3105068

[jkae057-B33] Lee YS, Nakahara K, Pham JW, Kim K, He Z, Sontheimer EJ, Carthew RW. 2004. Distinct roles for Drosophila dicer-1 and dicer-2 in the siRNA/miRNA silencing pathways. Cell. 117(1):69–81. doi:10.1016/s0092-8674(04)00261-2. 15066283

[jkae057-B34] Legube G, McWeeney SK, Lercher MJ, Akhtar A. 2006. X-chromosome-wide profiling of MSL-1 distribution and dosage compensation in Drosophila. Genes Dev. 20(7):871–883. doi:10.1101/gad.377506. 16547175 PMC1472288

[jkae057-B35] Lim DH, Kim J, Kim S, Carthew RW, Lee YS. 2008. Functional analysis of dicer-2 missense mutations in the siRNA pathway of Drosophila. Biochem Biophys Res Commun. 371(3):525–530. doi:10.1016/j.bbrc.2008.04.118. 18454937 PMC2924196

[jkae057-B36] Lucchesi JC, Kelly WG, Panning B. 2005. Chromatin remodeling in dosage compensation. Annu Rev Genet. 39(1):615–651. doi:10.1146/annurev.genet.39.073003.094210. 16285873

[jkae057-B37] Lucchesi JC, Kuroda MI. 2015. Dosage compensation in Drosophila. Cold Spring Harb Perspect Biol. 7(5):a019398. doi:10.1101/cshperspect.a019398. 25934013 PMC4448616

[jkae057-B38] Meller VH, Rattner BP. 2002. The roX genes encode redundant male-specific lethal transcripts required for targeting of the MSL complex. EMBO J. 21(5):1084–1091. doi:10.1093/emboj/21.5.1084. 11867536 PMC125901

[jkae057-B39] Meller VH, Wu KH, Roman G, Kuroda MI, Davis RL. 1997. Rox1 RNA paints the X chromosome of male Drosophila and is regulated by the dosage compensation system. Cell. 88(4):445–457. doi:10.1016/s0092-8674(00)81885-1. 9038336

[jkae057-B40] Menon DU, Coarfa C, Xiao W, Gunaratne PH, Meller VH. 2014. siRNAs from an X-linked satellite repeat promote X-chromosome recognition in Drosophila melanogaster. Proc Natl Acad Sci USA. 111(46):16460–16465. doi:10.1073/pnas.1410534111. 25368194 PMC4246271

[jkae057-B41] Menon DU, Meller VH. 2012. A role for siRNA in X-chromosome dosage compensation in Drosophila melanogaster. Genetics. 191(3):1023–1028. doi:10.1534/genetics.112.140236. 22554892 PMC3389965

[jkae057-B42] Oh H, Park Y, Kuroda MI. 2003. Local spreading of MSL complexes from roX genes on the Drosophila X chromosome. Genes Dev. 17(11):1334–1339. doi:10.1101/gad.1082003. 12782651 PMC196065

[jkae057-B43] Öztürk-Çolak A, Marygold SJ, Antonazzo G, Attrill H, Goutte-Gattat D, Jenkins VK, Matthews BB, Millburn G, Dos Santos G, Tabone CJ. 2024. FlyBase: updates to the Drosophila genes and genomes database. Genetics. iyad211. doi:10.1093/genetics/iyad211. 38301657 PMC11075543

[jkae057-B44] Park Y, Kelley RL, Oh H, Kuroda MI, Meller VH. 2002. Extent of chromatin spreading determined by roX RNA recruitment of MSL proteins. Science (New York, N.Y.). 298(5598):1620–1623. doi:10.1126/science.1076686. 12446910

[jkae057-B45] Park Y, Mengus G, Bai X, Kageyama Y, Meller VH, Becker PB, Kuroda MI. 2003. Sequence-specific targeting of Drosophila roX genes by the MSL dosage compensation complex. Mol Cell. 11(4):977–986. doi:10.1016/s1097-2765(03)00147-3. 12718883

[jkae057-B46] Park SW, Oh H, Lin YR, Park Y. 2010. MSL cis-spreading from roX gene up-regulates the neighboring genes. Biochem Biophys Res Commun. 399(2):227–231. doi:10.1016/j.bbrc.2010.07.059. 20654579

[jkae057-B47] Pirrotta V, Steller H, Bozzetti MP. 1985. Multiple upstream regulatory elements control the expression of the Drosophila white gene. EMBO J. 4(13A):3501–3508. doi:10.1002/j.1460-2075.1985.tb04109.x. 3004963 PMC554689

[jkae057-B48] Ramírez F, Lingg T, Toscano S, Lam KC, Georgiev P, Chung HR, Lajoie BR, de Wit E, Zhan Y, de Laat W, et al 2015. High-Affinity sites form an interaction network to facilitate spreading of the MSL Complex across the X chromosome in Drosophila. Mol Cell. 60(1):146–162. doi:10.1016/j.molcel.2015.08.024. 26431028 PMC4806858

[jkae057-B49] Schotta G, Ebert A, Krauss V, Fischer A, Hoffmann J, Rea S, Jenuwein T, Dorn R, Reuter G. 2002. Central role of Drosophila SU(VAR)3-9 in histone H3-K9 methylation and heterochromatic gene silencing. EMBO J. 21(5):1121–1131. doi:10.1093/emboj/21.5.1121. 11867540 PMC125909

[jkae057-B50] Smith ER, Pannuti A, Gu W, Steurnagel A, Cook RG, Allis CD, Lucchesi JC. 2000. The Drosophila MSL complex acetylates histone H4 at lysine 16, a chromatin modification linked to dosage compensation. Mol Cell Biol. 20(1):312–318. doi:10.1128/MCB.20.1.312-318.2000. 10594033 PMC85086

[jkae057-B51] Soruco MM, Chery J, Bishop EP, Siggers T, Tolstorukov MY, Leydon AR, Sugden AU, Goebel K, Feng J, Xia P, et al 2013. The CLAMP protein links the MSL complex to the X chromosome during Drosophila dosage compensation. Genes Dev. 27(14):1551–1556. doi:10.1101/gad.214585.113. 23873939 PMC3731544

[jkae057-B52] Spierer A, Begeot F, Spierer P, Delattre M. 2008. SU(VAR)3-7 links heterochromatin and dosage compensation in Drosophila. PLoS Genet. 4(5):e1000066. doi:10.1371/journal.pgen.1000066. 18451980 PMC2320979

[jkae057-B53] Spierer A, Seum C, Delattre M, Spierer P. 2005. Loss of the modifiers of variegation Su(var)3-7 or HP1 impacts male X polytene chromosome morphology and dosage compensation. J Cell Sci. 118(21):5047–5057. doi:10.1242/jcs.02623. 16234327

[jkae057-B54] Straub T, Grimaud C, Gilfillan GD, Mitterweger A, Becker PB. 2008. The chromosomal high-affinity binding sites for the Drosophila dosage compensation complex. PLoS Genet. 4(12):e1000302. doi:10.1371/journal.pgen.1000302. 19079572 PMC2586088

[jkae057-B55] Sural TH, Peng S, Li B, Workman JL, Park PJ, Kuroda MI. 2008. The MSL3 chromodomain directs a key targeting step for dosage compensation of the Drosophila melanogaster X chromosome. Nat Struct Mol Biol. 15(12):1318–1325. doi:10.1038/nsmb.1520. 19029895 PMC2636508

[jkae057-B56] Waring GL, Pollack JC. 1987. Cloning and characterization of a dispersed, multicopy, X chromosome sequence in Drosophila melanogaster. Proc Natl Acad Sci USA. 84(9):2843–2847. doi:10.1073/pnas.84.9.2843. 3106978 PMC304756

