# Supplementary material for: Identification of X chromatin is modulated by complementary pathways in Drosophila melanogaster
Source: G3 (Bethesda). 2024 Mar 16;14(6):jkae057. doi: 10.1093/g3journal/jkae057 (PMC11152068; doi:10.1093/g3journal/jkae057)
Supplement: jkae057_Supplementary_Data [file jkae057_supplementary_data.docx]

**Supplemental Material**

**
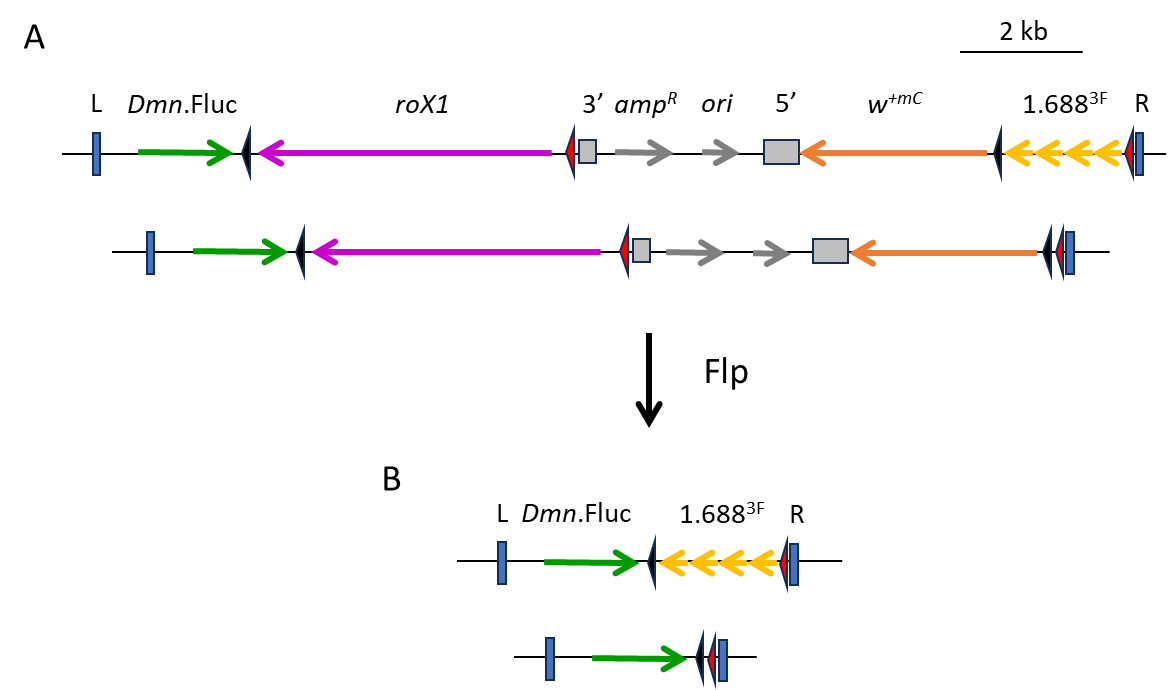
**

**Figure S1.** **Structure of the Dmn.Fluc transgene and reduced derivatives.**

A) The full transgene following integration into the genome (top) and a version deleted for the 1.688^3F^ repeats (bottom). From left to right: L, attL; Dmn-driven Fluc flanked by *msl2* 5' and 3' UTRs (green arrow); FRT (black triangle); 4.9 kb genomic fragment containing *roX1* (purple); LoxP (red triangle); 3’ P-end (gray box); amp^R^ and ori (gray arrows); 5’ P-end (gray box); *w^+mC^* marker (orange arrow); FRT; 1.688^3F^ repeats (yellow arrows); LoxP; attR. B) Reduction of the full transgene with Flp removes *roX*1, amp^R^, ori and *w^+mC^* (top). Reduction of the transgene lacking repeats (bottom), produces a Dmn.Fluc control with no recruiting elements.


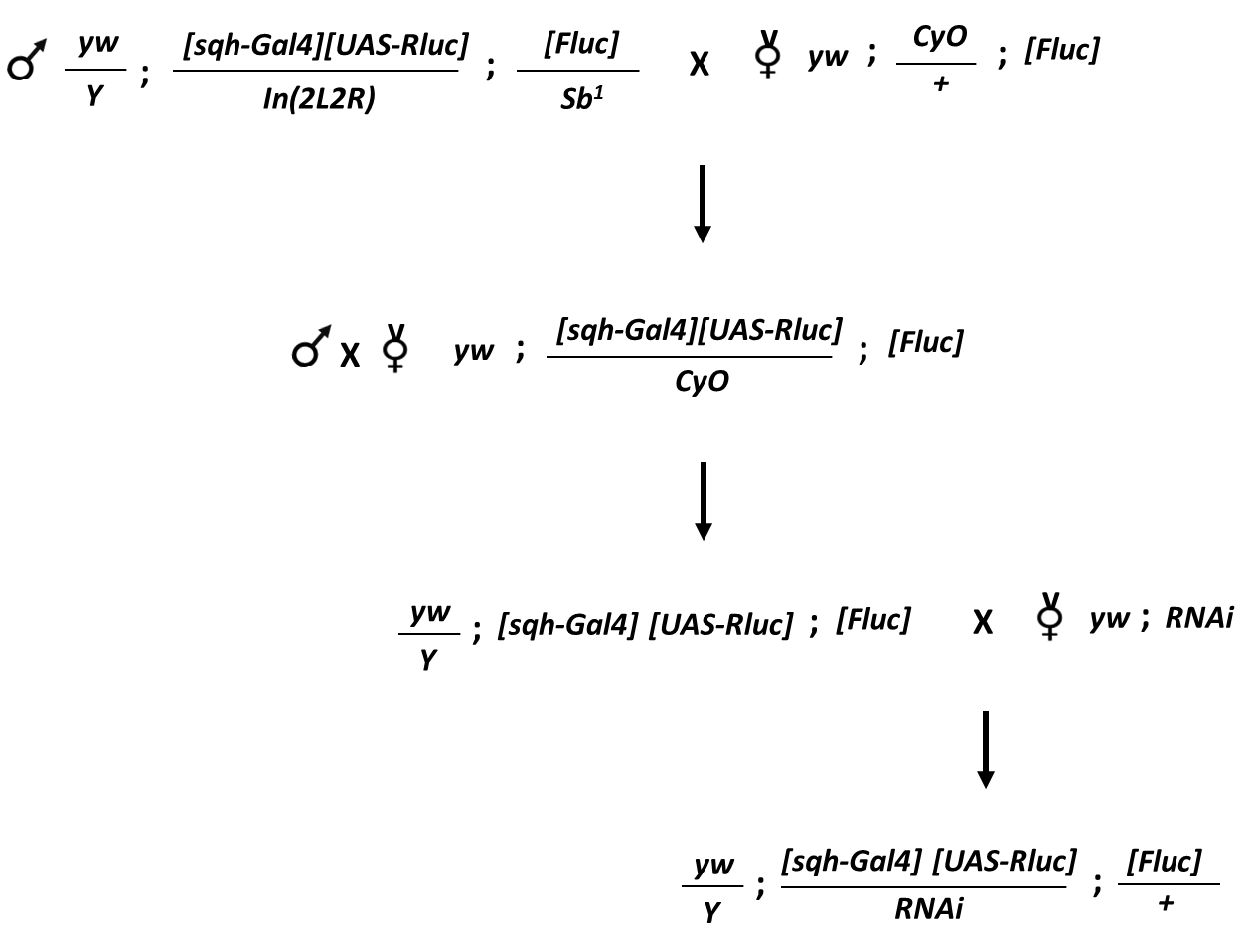


**Figure S2. Mating scheme to generate RNAi knockdown in the different reporter constructs.**


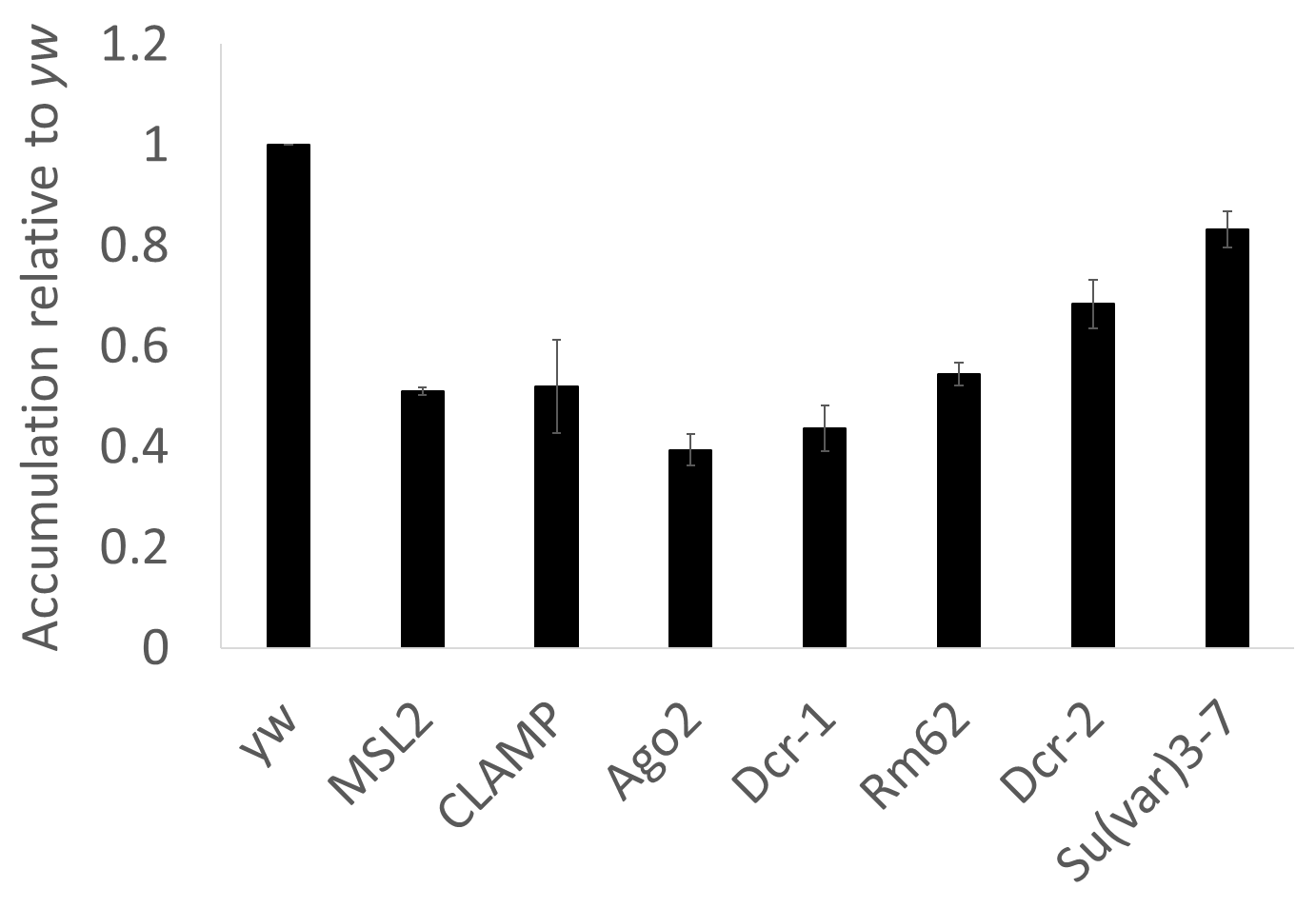


**Figure S3. mRNA accumulation upon knock down.**

Normalized mRNA accumulation following RNAi knock down for lines used in this study. Expression relative to the laboratory reference *yw* strain is normalized to *Dmn*. MSL2, CLAMP, Ago2 and Dcr-1 validation was performed on RNA from 3^rd^ instar larvae. Rm62, Dcr-2 and Su(var)3-7 validation was performed using RNA isolated from embryos. RNAi lines and the P{sqh-GAL4}2 driver used are presented in Table S1. Error bars represent SEM of three biological replicates.


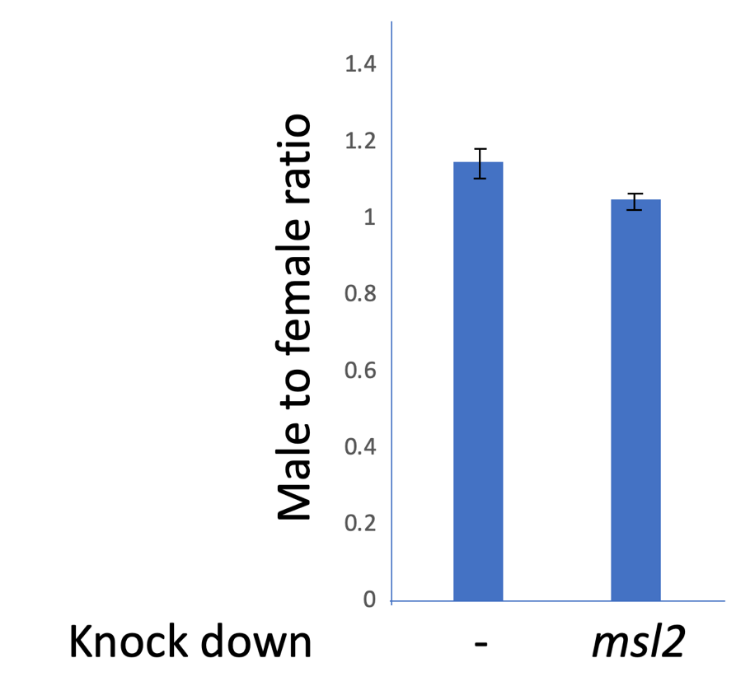


**Figure S4. Knock down of *msl2* does not skew embryonic sex ratios.**

Collections of 6-12 hour embryos carrying Sxl-Pe LacZ and driving RNAi for MSL2 knock down were stained with X-gal and scored. All embryos with B-galactosidase activity were classed as female. Bars represent the mean and standard deviation of two replicate counts. Full genotype is: *yw*; P{ w^+mC^  Sxl Pe-LacZ} P{sqh-GAL4}2/+; P{y[+t7.7] v[+t1.8]=TRiP.JF01412}attP2/+ **
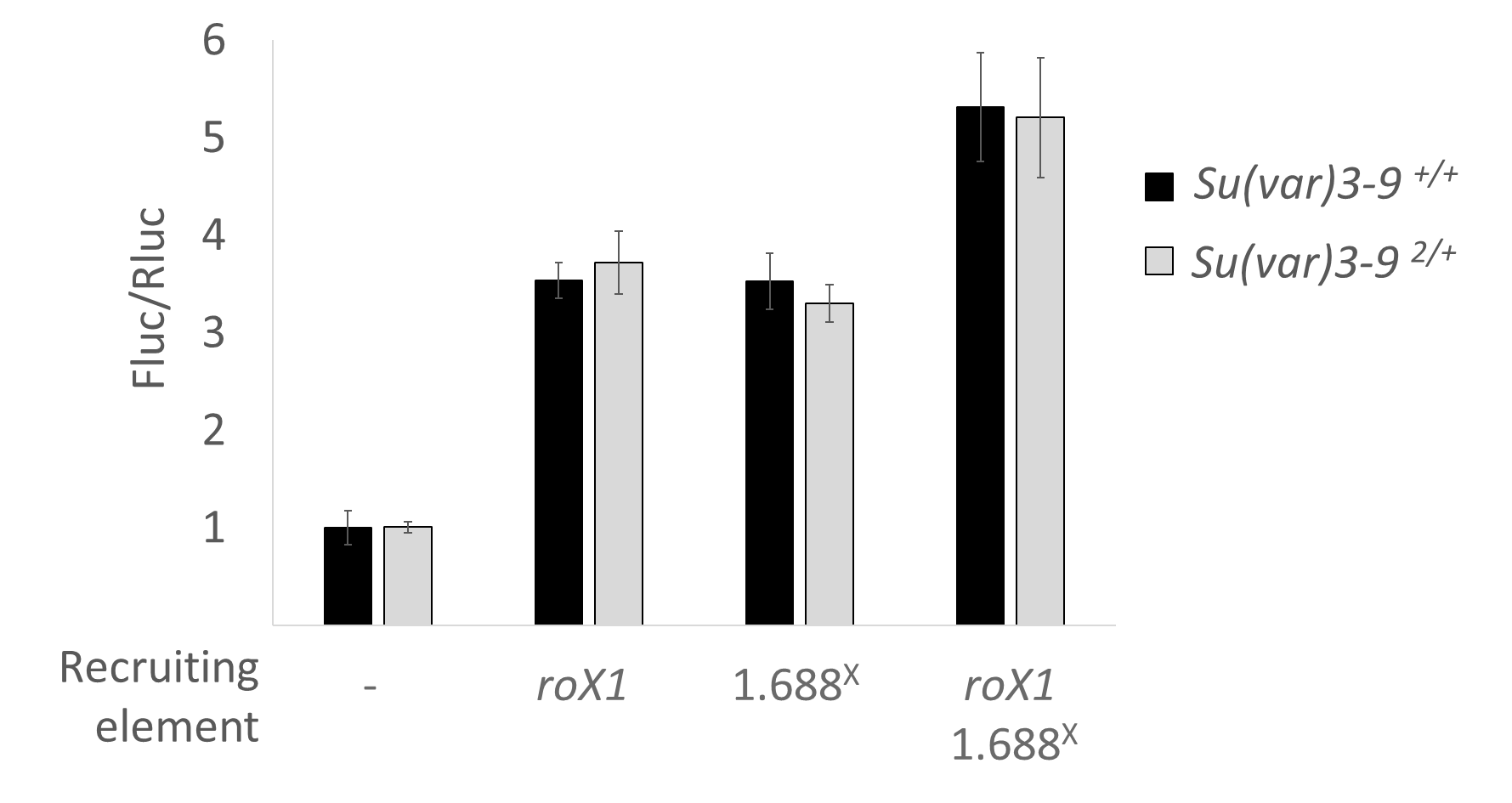
**

**Figure S5. Reduction of Su(var)3-9 does not affect the *Dmn*.Fluc reporter.**

*Dmn***.**Fluc activity is unaffected in *Su(var)3-9^2/+^* adult males (gray). Control flies (black) are wild type for *Su(var)3-9*. Error bars represent SEM of three biological replicates.

**Table S1. *Drosophila melanogaster* strains used in this study**

| **Name** | **Stock #, Supplier** | **Genotype** | **Flybase ID** |
| --- | --- | --- | --- |
| PBac at 65B2 | 9750 (BDSC) | y[1] w[1118]; PBac{y[+]-attP-3B}VK00033 | FBti0076453 |
| Rluc | 64774 (BDSC) | y[1] w[*]; M{w[+mC]=UAS-Rren\LUC.G}ZH-51C | FBti0181924 |
| Clamp RNAi | 57163 (BDSC) | y[1] sc[*] v[1] sev[21]; P{y[+t7.7] v[+t1.8]=TRiP.HMC04544}attP40 | FBgn0032979 |
| Msl2 RNAi | 31627 (BDSC) | y[1] v[1]; P{y[+t7.7] v[+t1.8]=TRiP.JF01412}attP2 | FBgn0005616 |
| Ago2 RNAi | 100356 (VDRC) | P{KK106052}VIE-260B | FBst0472229 |
| Dcr-1 RNAi | 28598 (BDSC) | y[1] v[1]; P{y[+t7.7] v[+t1.8]=TRiP.HM05086}attP2 | FBgn0039016 |
| Rm62 RNAi | 31395 (BDSC) | y[1] v[1]; P{y[+t7.7] v[+t1.8]=TRiP.JF01385}attP2 | FBgn0003261 |
| Dcr-2 RNAi | 33656 (BDSC) | y[1] sc[*] v[1] sev[21]; P{y[+t7.7] v[+t1.8]=TRiP.HMS00062}attP2 | FBgn0034246 |
| Su(var)3-7 RNAi | 64598 (BDSC) | y[1] sc[*] v[1] sev[21]; P{y[+t7.7] v[+t1.8]=TRiP.HMC05633}attP40 | FBgn0003598 |
| yellow RNAi | 64527 (BDSC) | y[1] sc[*] v[1] sev[21]; P{y[+t7.7] v[+t1.8]=TRiP.HMC05546}attP40 | FBgn0004034 |
| mCherry RNAi | 35785 (BDSC) | y[1] sc[*] v[1] sev[21]; P{y[+t7.7] v[+t1.8]=VALIUM20-mCherry.RNAi}attP2 | FBti0143385 |
| Su(var)3-9^2^ | 6210 (BDSC) | In(1)w[m4]; Su(var)3-9[2]/TM3, Sb[1] Ser[1] | FBal0016558 |
| P{sqh-GAL4}2 | Gift of S. Todi | P{w^+mC^sqh-GAL4}2 | FBti0074562 |
| Sxl Pe-LacZ | Gift of P. Schedl | P{ w^+mC^  Sxl Pe-LacZ} | FBal0041988 |
